# The effect of nanoemulsified methionine and cysteine on the *in vitro* expression of casein in bovine mammary epithelial cells

**DOI:** 10.5713/ajas.18.0203

**Published:** 2018-07-26

**Authors:** Tae-Il Kim, Tae-Gyun Kim, Dong-Hyun Lim, Sang-Bum Kim, Seong-Min Park, Hyun-Joo Lim, Hyun-Jong Kim, Kwang-Seok Ki, Eung-Gi Kwon, Young-Jun Kim, Vijayakumar Mayakrishnan

**Affiliations:** 1 Dairy Science Division, National Institute of Animal Science, Rural Development Administration, Cheonan 31000, Korea; 2 Department of Food and Biotechnology, Korea University, Sejong 30019, Korea; 3 Hanwoo Research Institute, National Institute of Animal Science, Rural Development Administration, Pyeongchang 25340, Korea

**Keywords:** Casein, Amino Acids, Methionine, Cysteine, Mammary Epithelial Cells

## Abstract

**Objective:**

Dairy cattle nutrient requirement systems acknowledge amino acid (AAs) requirements in aggregate as metabolizable protein (MP) and assume fixed efficiencies of MP used for milk protein. Regulation of mammary protein synthesis may be associated with AA input and milk protein output. The aim of this study was to evaluate the effect of nanoemulsified methionine and cysteine on the *in-vitro* expression of milk protein (casein) in bovine mammary epithelial cells (MAC-T cells).

**Methods:**

Methionine and cysteine were nonionized using Lipoid S 75 by high-speed homogenizer. The nanoemulsified AA particle size and polydispersity index were determined by dynamic light scattering correlation spectroscopy using a high-performance particle sizer instrument. 3-(4,5-Dimethylthiazol-2-yl)-2,5-diphenyltetrazolium bromide assay was performed to determine the cytotoxicity effect of AAs with and without nanoionization at various concentrations (100 to 500 μg/mL) in mammary epithelial cells. MAC-T cells were subjected to 100% of free AA and nanoemulsified AA concentration in Dulbecco’s modified Eagle medium/nutrient mixture F-12 (DMEM/F12) for the analysis of milk protein (casein) expression by the quantitative reverse transcription polymerase chain reaction method.

**Results:**

The AA-treated cells showed that cell viability tended to decrease (80%) in proportion to the concentration before nanogenesis, but cell viability increased as much as 90% after nanogenesis. The analysis of the expression of genetic markers related to milk protein indicated that; α_s2_-casein increased 2-fold, κ-casein increased 5-fold, and the amount of unchanged β-casein expression was nearly doubled in the nanoemulsified methionine-treated group when compared with the free-nanoemulsified methionine-supplemented group. On the contrary, the non-emulsified cysteine-administered group showed higher expression of genetic markers related to milk protein α_s2_-casein, κ-casein, and β-casein, but all the genetic markers related to milk protein decreased significantly after nanoemulsification.

**Conclusion:**

Detailed knowledge of factors, such nanogenesis of methionine, associated with increasing cysteine and decreasing production of genetic markers related to milk protein (casein) will help guide future recommendations to producers for maximizing milk yield with a high level of milk protein casein.

## INTRODUCTION

Dairy cattle nutrient requirement systems consider amino acids (AA) requirements to be essential in aggregate as metabolizable protein (MP) and assume fixed efficiencies of MP used for milk protein. They are used to determine the amount of absorbed AAs or nitrogen (N) required to support a preferred level of milk production with milk protein [1]. The AA composition affects the synthesis of milk protein. The exact amount of limited AA in dairy cow diets is unknown, and cows are overfed to meet the equilibrium of MP needed. This leads to AA waste and poor N efficiency [2]. In the previous literature, approximately 25% of dietary N is captured in milk, and the remaining approximately 75% is captured in urine and feces of dairy cows [3]. Therefore, feeding cows with a low protein concentration of essential and non-essential AA supplements may maintain and improve the total N efficiency [4–6]. In lactating dairy cows, AAs play a significant role in mammary protein synthesis. The previous literature on AA metabolism has mainly focused on balanced diets, which are necessary to maintain and enhance milk protein synthesis in lactating cows [7]. In mammals, AAs not only work as precursors for the synthesis of milk protein but also as signaling molecules for the regulation of protein synthesis and lactation [8]. For example, Moshel et al [9] reported that leucine supplementation increases the β-lactoglobulin synthesis in mouse and bovine mammary epithelial cells. Furthermore, isoleucine, methionine and threonine affect milk protein (casein) synthesis when removed from the culture medium of bovine mammary epithelial cells. So, a sufficient supply of AAs can increase the MP of milk protein and milk yield. Numerous studies have shown that AAs stimulate the synthesis of milk protein, which is mediated by mammalian target of rapamycin (mTOR) [10]. Histidine was the first AA found for the synthesis of milk protein [4]. Previous literature also demonstrated that introduction of histidine enhanced milk protein secretion [11]. A study by Appuhamy et al [6] showed that the addition of histidine to the cells activated the mTOR pathway and promoted milk protein synthesis, which upregulated phosphorylation of the downstream protein. Similarly, histidine was one of the transcriptional factors of the mTORC1 pathway, and it was negatively regulated by phosphorylated S6 kinase 1 and decreased the β-casein synthesis rate [12]. The consequences of essential AAs on milk protein synthesis and the mTOR signaling pathway have been extensively studied [13].

Nanoemulsification is a promising technology that has been used in various fields, including the pharmaceutical and food industries, with several potential applications. It is defined as a process by which substances of core materials are surrounded by a wall material to obtain capsules [14]. Liposomes are nano-sized vesicles consisting of a membrane-like phospholipid bilayer surrounding an aqueous medium. Liposomes have been widely used in the pharmaceutical, food, and cosmetic industries, and have been successfully employed for the encapsulation of a wide range of synthetic drugs and bioactive molecules [15,16]. The application of liposomes onto bioactive compounds at the nanoscale range protects them from chemical degradation by the surrounding dispersion medium. Encapsulation implements the controlled release of bioactive molecules at the right place and time and it also increases the shelf life of bioactive molecules. Liposomes can be prepared as Lipoid S 75 (phosphatidylcholine [PC]>75%) from soybean lecithin. Amphiphilic lipoid S 75 is composed of a hydrophilic head domain and hydrophobic tail domain. This can come from the liposome in the aqueous media having a structure of a hydrophilic surface and a hydrophobic inner layer. It would be an ideal core material to prepare carriers for a nutrient which has the biological activity itself [17]. To the best of our knowledge, there is no scientific data on the effect of nanoemulsified AAs in casein expression. Therefore, the purpose of this experiment was to determine the effect of liposome-coated methionine and cysteine on the *in vitro* expression of milk protein casein in bovine mammary epithelial cells.

## MATERIALS AND METHODS

### Cell culture and chemicals

The mammary epithelial (MAC-T) cell line (ATCC: CRL-10274) was procured from the American Type Culture Collection (Rockville, MD, USA). Dulbecco’s modified Eagle medium (DMEM), fetal bovine serum (FBS), penicillin, streptomycin, gentamicin, and hydrocortisone were purchased from Gibco-BRL (Gaithersburg, MD, USA). The kit for mRNA isolation and reverse transcription polymerase chain reaction (RT-PCR) kit were procured from Invitrogen (Carlsbad, CA, USA). 3-(4,5-Dimethylthiazol-2-yl)-2,5-diphenyltetrazolium bromide (MTT) was obtained from Amresco (Solon, OH, USA). Insulin was purchased from Sigma Aldrich (St. Louis, MO, USA). All other chemicals and solvents were of analytical grade. Lipoid S 75, Lipoid S 100, Lecinol S 10, Hydrogenated soybean phosphatidylcholine (HSPC) 50, Lipoid S 75-3, and Lipoid S 100-3 were purchased from Pharmachem (Seoul, Korea).

### Production of nanoemulsion for amino acids delivery

The AAs were encapsulated in lecithin (Lipoid S 75)-based nanoemulsions, produced by high-pressure homogenization (HPH), as described by a previous study. Briefly, 10 wt. % of AAs were diluted with 10 wt. % of ethanol, and then the mixture was added to 5 wt. % of lecithin, 75 wt. % of water and stirred by blender (develop pre-emulsion) until completely emulsified by high-speed homogenization using an Ultra Turrax T25 blender (IKA Labortechnik, Germany) for 4 min at 24,000 rpm, to achieve a primary emulsion. After that, cooled pre-emulsion was passed through a high-pressure homogenizer (MN400BF, Micronox, Seongnam, Korea) 3 times at 1,000 psi, resulting in nanoemulsion. The collected nanoemulsified AAs (methionine and cysteine) were used for further analysis.

### Particle size and polydispersity analysis

The particle size distribution and polydispersity (zeta potential) of the nanoemulsified methionine and cysteine were analyzed by dynamic light scattering correlation spectroscopy using a high-performance particle sizer instrument (Malvern Instruments, Malvern, UK). The droplet size distribution was characterized regarding the mean droplet size (Z-diameter) and width of the distribution (polydispersity index, PDI) at a scattering angle of 190° at 25°C. Briefly, by measuring the backscattered light of 100 μL samples diluted in 1 mL of distilled water put into a polystyrene latex cell and measured at a scattering angle of 90°, dispersant refractive index of 1.33, and material refractive index of 1.59 at 25°.

### Cytotoxicity of nanoemulsions

MTT was used in cytotoxicity analysis. Briefly, cells were seeded in a 96-well plate at a density of 0.5×10^4^ cells/well. After a 24-h interval, cells were treated with different concentrations (100 to 500 μg/mL) of nanoemulsified AAs (methionine and cysteine). After 48 h of incubation at 37°C with 5% CO_2_, cells were treated with 10 μL of MTT solution and incubated for 4 h. The optical density of each well was measured at a wavelength of 540 nm using a Spectra count enzyme-linked immunosorbent assay plate reader (Packard Instrument Co., Downers Grove, IL, USA). The cytotoxicity of the blank nano-encapsulated AAs was expressed as the percentage of cell viability, calculated from the ratio between the numbers of living cells treated with the nano-encapsulated AAs and that of the untreated cells. Based on the cytotoxicity data, the concentration of encapsulated AAs (18 μg/mL) was chosen for the present study.

### Cell culture and treatment

Bovine mammary epithelial cells were cultured in a 12-well plate at a density of 1.5×10^4^ cells/well in DMEM supplemented with 10% of FBS, 100 μg/mL of penicillin and streptomycin, 5 μg/mL of insulin, 50 μg/mL of gentamicin, and 1 μg/mL hydrocortisone in a humidified atmosphere, including 5% CO_2_ at 37°C. After cells reached approximately 90% to 100% confluency, they were treated with nanoemulsified AAs (methionine and cysteine) at a concentration of 18 μg/mL for 24 h. The experiment performed in triplicate.

### Milk protein gene expression quantification by quantitative reverse transcription polymerase chain reaction

At the end of the experiment, cellular mRNA was extracted from MAC-T cells by a RNeasy lipid tissue kit (Qiagen, Valencia, CA, USA) according to the manufacturer’s protocol. Extracted RNA was quantified with UVS-99 microvolume UV/Vis spectrometer-ACT gene. cDNA synthesis was performed with 500 ng of cellular RNA using oligo (dT) primers and reverse transcriptase provided by the Superscript III first-strand synthesis system for RT-PCR (Invitrogen, USA). The level of casein-related gene mRNA expression was assessed by SYBR Green-based real-time PCR on an ABI 7500 PCR system (Applied Biosystem, Foster City, CA, USA). The target gene expression levels were normalized against housekeeping gene glyceraldehyde 3-phosphate dehydrogenase. Primers used for quantitative RT-PCR are listed in Table 1.

### Statistical analysis

All the experiments were conducted in triplicate. Data were statistically analyzed, and comparison was performed using a statistical package (SPSS-16.0) (SPSS, Inc., Chicago, IL, USA). The results were represented as a mean±standard error of the mean. The significant difference between the mean was compared by least significant difference, and the value was considered p<0.05.

## RESULTS AND DISCUSSION

### Characterization of AA nanoemulsion

**Figure 1:**
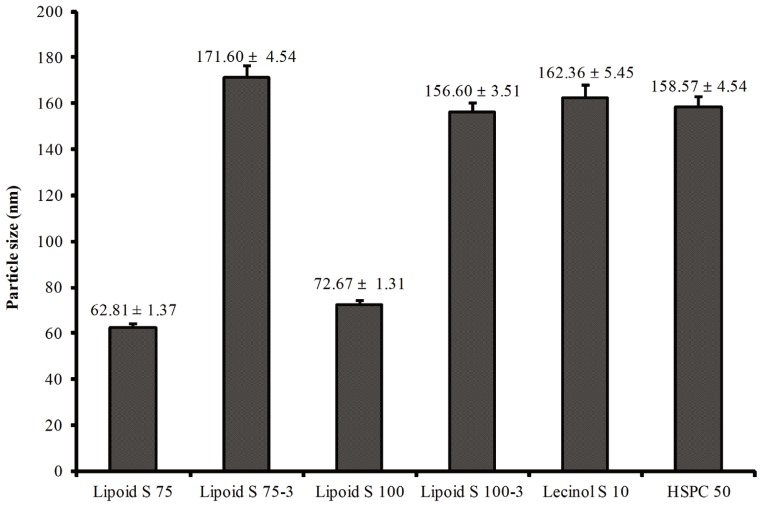
Particle size of core materials analyzed by dynamic light scattering correlation spectroscopy. The core material of Lipoid S 75 had greater efficiency and a smaller particle size when compared to other core materials of Lipoid S 75-3, S 100, S 100-3, Lecinol S 10, and HSPC 50. HSPC, hydrogenated soybean phosphatidylcholine.

**Figure 2:**
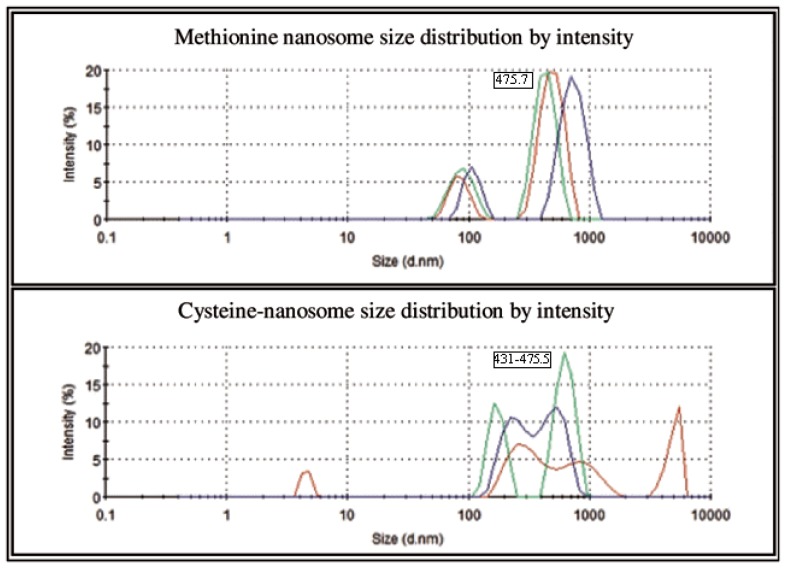
Particle size distribution of nanoemulsified methionine and cysteine nanosomes by intensity using dynamic light scattering correlation spectroscopy. The average particle size distribution of nanoemulsion of methionine in Lipoid S 75 was in the range of 475.7 d.nm, whereas, the particle size distribution of the cysteine in Lipoid S 75 in the range of 431 to 475 d.nm by intensity.

**Figure 3:**
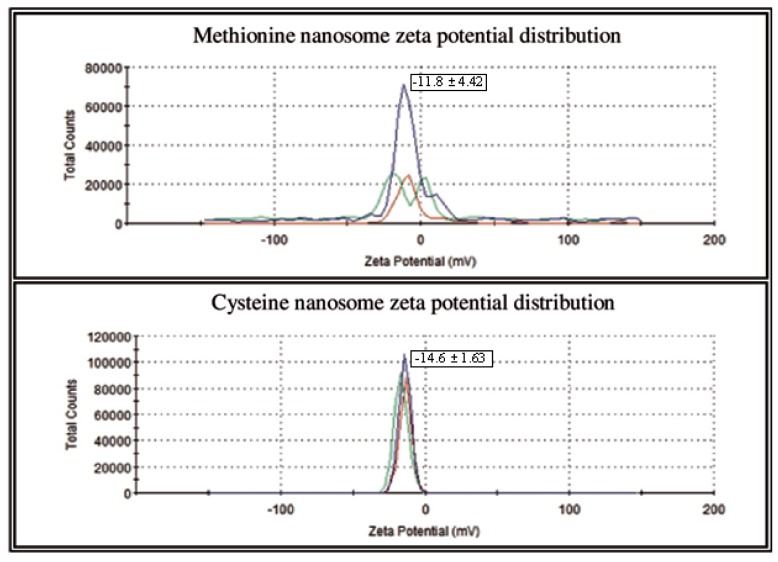
Zeta potential distribution of nanoemulsified methionine and cysteine nanosomes by intensity using dynamic light scattering correlation spectroscopy. The zeta potential of nanoemulsion of methionine and cysteine in Lipoid S 75 was −11.8±349 4.42 mV and −14.6±1.63 mV, respectively.

The effectiveness of the core material used to prepare AA nanoemulsion was determined from the total amount of AAs in nanoemulsions and AAs that were extracted from surface of nano particles. The core material of Lipoid S 75 had greater efficiency and a smaller particle size (62.81±1.37 nm) when compared to other core materials of Lipoid S 75-3 (171.60±4.54 nm), Lipoid S 100 (72.67±1.31 nm), Lipoid S 100-3 (156.60± 3.51 nm), Lecinol S 10 (162.36±5.45 nm), and HSPC 50 (158.57 ±4.54 nm) (Figure 1). Based on these properties, we selected Lipoid S 75 for the preparation of nanoemulsified AAs. Methionine with Lipoid S 75 was entrapped in unsaturated soybean lecithin with an encapsulation efficiency of 77.6%. Similarly, cysteine with Lipoid S 75 was entrapped in unsaturated soybean lecithin with an encapsulation efficiency of 89.8%. The average particle size distribution of nanoemulsion of methionine in Lipoid S 75 was in the range of 475.7 nm, whereas, the particle size distribution of the cysteine in Lipoid S 75 was in the range of 431 to 475 nm by intensity as presented in Figure 2. The results of the current study showed that high-pressure homogenization 3 times at 1,000 psi was the optimal condition to prepare an AA nanoemulsion. The zeta potential of nanoemulsion of methionine and cysteine in Lipoid S 75 was −11.8±4.42 mV and −14.6±1.63 mV, respectively (Figure 3). The high PDI was caused by the main limitation of dynamic light scattering due to high concentration in the cuvette. This can lead to Brownian motion, such as multiple scattering with a decreased path length of particles. These phenomena can result in unfavorable particle size. Dynamic light scattering measures the hydrodynamic radius of a particle. A possible reason may be that Lipoid S 75 can attract water as more water is added; therefore, the dynamic diameter increases slightly. As the sample becomes more diluted, more Lipoid S 75 may move out of the particle, causing the particle size to decrease. Fortunately, this was not the case, as this can give substantial problems when this would be used for therapeutic applications [18]. In addition, all further studies of cytotoxicity and efficacy using methionine and cysteine in Lipoid S 75 on milk protein in MAC-T cells were performed based on the efficiency and particle size of nanoemulsion-based delivery systems.

### Cytotoxicity of nanosomes

Colorimetric MTT assays were performed 24 h after treatment with or without nanoemulsified of AAs to highlight a possible cytotoxic effect of these emulsions (Table 2). Results of the present study showed that cell viability was slightly agitated at AA concentrations up to 500 μg/mL compared with control cells (p<0.05). Findings of the current study support those of Basirico et al [19], who previously reported that no fatty acid treatment on mammary epithelial cells reduced cell viability. However, at the same time, nanoemulsified AAs significantly increased the cell viability at concentrations up to 500 μg/mL when compared with non-emulsified AAs (p<0.05). Although the AAs had no significant effect regardless of the dose used, the dose-response curve showed that the administration of 100 to 500 μg/mL of nanoemulsified AAs increased the cell viability from 10.1% to 11.9%, respectively when compared with free AAs.

### Effects of methionine and cysteine nanoemulsion on the expression of caseins

**Figure 4:**
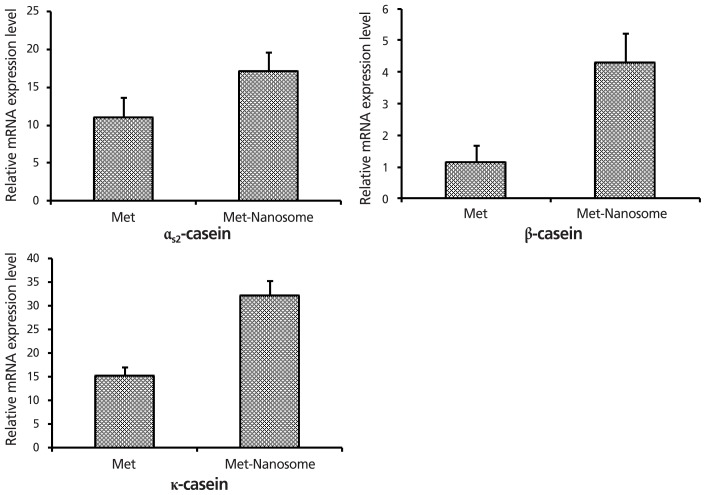
Effect of free-methionine and nanoemulsified methionine on casein gene expression in bovine mammary epithelial cells analyzed by quantitative reverse transcription polymerase chain reaction. The nanoemulsified methionine had stimulatory effects of 2 fold on the α_s2_-casein, 5 fold on the β-casein, and 2 fold on the κ-casein expression when compared with the non-emulsified methionine group.

**Figure 5:**
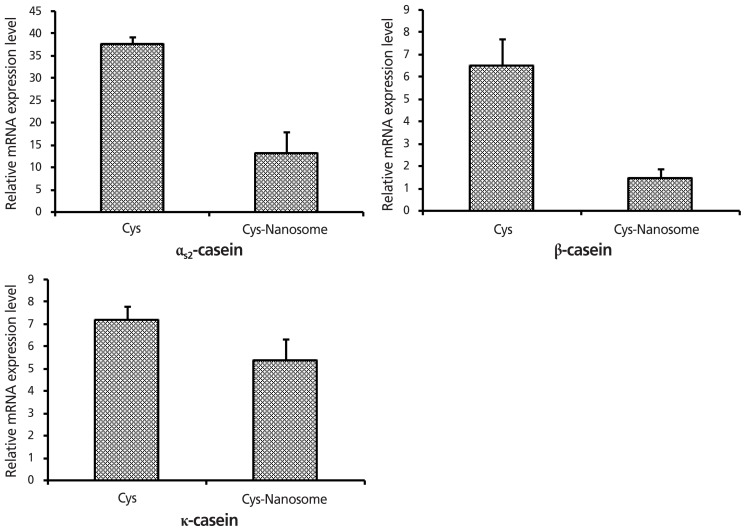
Effect of free-cysteine and nanoemulsified cysteine on casein gene expression in bovine mammary epithelial cells analyzed by quantitative reverse transcription polymerase chain reaction. Compared with non-emulsified cysteine, the expression of α_s2_-casein, β-casein, and κ-casein was decreased by emulsified cysteine.

Several studies have evaluated the contribution of AAs to mammary gland metabolism and protein secretion and synthesis [20]. In this experiment, the potential effect of nanoemulsified methionine and cysteine on milk protein casein expression in bovine epithelial mammary cells was studied. The current study showed that, compared with the non-emulsified methionine group, the supplementation of methionine to Lipoid S 75 generated significantly increased effects on the casein expression. Figure 4 shows that nanoemulsified methionine had stimulatory effects of 2-fold on the α_s2_-casein, 5-fold on the β-casein, and 2-fold on the κ-casein expression when compared with the non-emulsified methionine group, which sustained the notion that methionine is a key limiting factor for milk protein synthesis. Our study results confirmed that nanoemulsified methionine promoted milk protein α-casein, β-casein, and κ-casein expression in cultured mammary epithelial cells as compared with non-emulsified methionine. Our current study findings agreed with those reported previously that histidine-containing dipeptide promoted milk protein production compared with supplementation of free histidine [21]. However, the mechanism of nanoemulsified AA utilization is complicated and unclear. It has been previously demonstrated that peptides containing methionine can easily utilize free methionine supplementation for mammary tissue and milk protein synthesis in cultured mammary epithelial cells [22]. A rat model experiment clearly showed that dipeptides were hydrolyzed extracellularly when compared with free AAs [23]. However, it remains unclear why supplementation with dipeptides containing methionine to cultured mammary epithelial cells significantly increased efficiency of α_s2_-casein gene expression, but α_s1_-casein gene expression was not increased when supplementation of free methionine was higher than 60 μg/mL in cultured bovine mammary epithelial cells [24]. The free methionine transporter may be saturated at 60 μg/mL, but dipeptides containing methionine may be absorbed through different transporters located on bovine mammary epithelial cell membranes [25]. This transporter may be from a different species, so bovine mammary epithelial cell membranes containing the transporter have a high affinity and absorb dipeptide methionine directly for milk protein casein synthesis. Figure 5 shows the effect of nanoemulsified cysteine supplementation on casein expression. Compared with non-emulsified cysteine, the expression of α_s2_-casein, β-casein, and κ-casein was decreased by emulsified cysteine. The cysteine content is lower in cow’s milk than in human milk because methionine content is higher in cow’s milk. Therefore, the methionine/cysteine ratio is 2- to 3-fold greater in cow’s milk than that of other mammals, but 7-fold greater in human milk. Two considerable characteristics of AA composition of human milk are the ratio between the sulfur-containing AAs methionine and cysteine, and the low level of aromatic AAs phenylalanine and tyrosine because of the low levels of the specific enzymes required to metabolize them. Wu et al [26] demonstrated that AAs, including arginine, cysteine, glutamine, and leucine act as both cell signaling candidates and regulators for gene expression and protein phosphorylation pathways. The expression of the α_s2_-casein, β-casein, and κ-casein genes was decreased when nanoemulsified cysteine was used to supplement the bovine mammary epithelial cells. Our study results agree with those of Raggio et al [27], who reported that the efficiency of the absorbed AAs into milk protein decrease markedly as protein delivery increases. However, further studies are needed to clarify the nature of the response by mammary cells to increase AA supply.

## CONCLUSION

In conclusion, the medium (soybean lecithin) used for mixing AAs (methionine and or cysteine) for emulsification enhanced encapsulation capability, size, and PDI. Addition of core material Lipoid S 75-based nanoemulsion of methionine to bovine mammary epithelial cells showed the potential to influence milk protein α_s2_-casein, β-casein, and κ-casein when compared with free methionine and cysteine. Therefore, further study is needed to determine how a nanoemulsion-based model may help to clarify the regulation mechanism of methionine and cysteine on casein expression through the mTOR pathway in bovine mammary epithelial cells.

## Figures and Tables

**Table 1:** Oligonucleotide primer sets for quantitative real-time polymerase chain reaction

Gene	Forward primer (5′-3′)	Reverse primer (3′-5′)	Source
*α*_s2_ *casein*	AGCTCTCCACCAGTGAGGAA	GCAAGGCGAATTTCTGGTAA	NM_174528.2
*β casein*	GTGAGGAACAGCAGCAGCAAACA	TTTTGTGGGAGGCTGTTAGG	NM_181008
*κ casein*	CCAGGAGCAAAACCAAGAAC	TGCAACTGGTTTCTGTTGGT	NM_174294
*GAPDH*	GGGTCATCATCTCTGCACCT	GGTCATAAGTCCCTCCACGA	XM_001252479

*GAPDH*, glyceraldehyde 3-phosphate dehydrogenase.

**Table 2:** Efficacy of methionine, methionine-nanosome, cysteine, and cysteine-nanosome in bovine mammary epithelial cell viability

Items	Methionine	Methionine-nanosome	Cysteine	Cysteine-nanosome
Control	100±0.15	100±0.16	100±0.15	100±0.12
100 μg/mL	89.21±0.77	94.38±1.35	93.16±0.84	100.06±0.98
200 μg/mL	88.17±0.39	91.78±2.92	90.42±1.49	98.42±1.51
300 μg/mL	85.37±2.46	91.30±2.32	89.89±2.35	93.93±1.10
400 μg/mL	81.64±1.35	88.89±4.27	89.39±3.59	93.34±0.76
500 μg/mL	80.46±3.37	88.71±3.52	89.21±2.96	90.81±1.19
